# Dye-loaded mesoporous polydopamine nanoparticles for multimodal tumor theranostics with enhanced immunogenic cell death

**DOI:** 10.1186/s12951-021-01109-7

**Published:** 2021-11-17

**Authors:** Ying Tian, Muhammad Rizwan Younis, Yuxia Tang, Xiang Liao, Gang He, Shouju Wang, Zhaogang Teng, Peng Huang, Longjiang Zhang, Guangming Lu

**Affiliations:** 1 Department of Medical Imaging, Jinling Hospital, Medical School of Nanjing University, Nanjing, 210002 People’s Republic of China; 2 State Key Laboratory of Analytical Chemistry for Life Science, School of Chemistry and Chemical Engineering, Nanjing University, Nanjing, 210093 People’s Republic of China; 3 Marshall Laboratory of Biomedical Engineering, International Cancer Center, Laboratory of Evolutionary Theranostics (LET), School of Biomedical Engineering, Shenzhen University Health Science Center, Shenzhen, 518060 People’s Republic of China; 4 Key Laboratory for Organic Electronics and Information Displays & Institute of Advanced Materials, Nanjing University of Posts & Telecommunications, Nanjing, 210023 People’s Republic of China

**Keywords:** Mesoporous polydopamine nanoparticles, IR-780, Photodynamic therapy, Photothermal therapy, Immunogenic cell death

## Abstract

**Background:**

Tumor phototherapy especially photodynamic therapy (PDT) or photothermal therapy (PTT), has been considered as an attractive strategy to elicit significant immunogenic cell death (ICD) at an optimal tumor retention of PDT/PTT agents. Heptamethine cyanine dye (IR-780), a promising PDT/PTT agent, which can be used for near-infrared (NIR) fluorescence/photoacoustic (PA) imaging guided tumor phototherapy, however, the strong hydrophobicity, short circulation time, and potential toxicity in vivo hinder its biomedical applications. To address this challenge, we developed mesoporous polydopamine nanoparticles (MPDA) with excellent biocompatibility, PTT efficacy, and PA imaging ability, facilitating an efficient loading and protection of hydrophobic IR-780.

**Results:**

The IR-780 loaded MPDA (IR-780@MPDA) exhibited high loading capacity of IR-780 (49.7 wt%), good physiological solubility and stability, and reduced toxicity. In vivo NIR fluorescence and PA imaging revealed high tumor accumulation of IR-780@MPDA. Furthermore, the combined PDT/PTT of IR-780@MPDA could induce ICD, triggered immunotherapeutic response to breast tumor by the activation of cytotoxic T cells, resulting in significant suppression of tumor growth in vivo.

**Conclusion:**

This study demonstrated that the as-developed compact and biocompatible platform could induce combined PDT/PTT and accelerate immune activation via excellent tumor accumulation ability, offering multimodal tumor theranostics with negligible systemic toxicity.

**Graphical Abstract:**

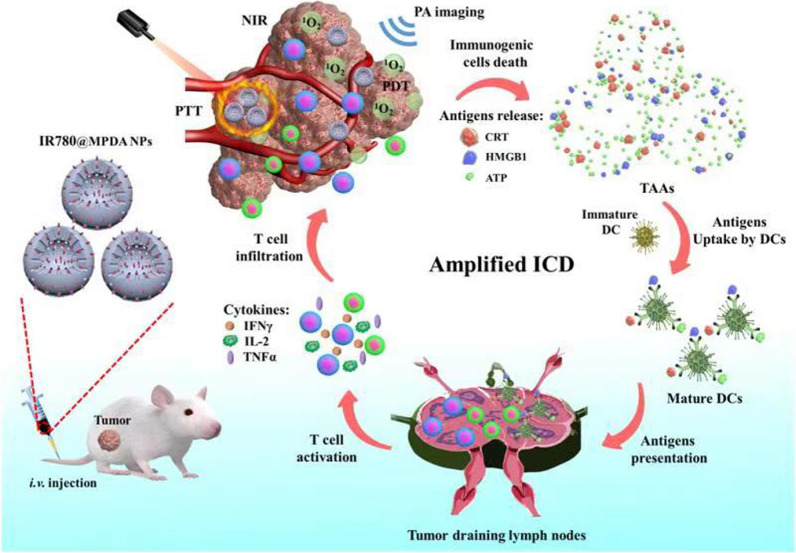

**Supplementary Information:**

The online version contains supplementary material available at 10.1186/s12951-021-01109-7.

## Introduction

Several anti-tumor nanomedicine strategies have been successfully developed, and among those, phototherapeutic tumor killing by photothermal therapy (PTT) or photodynamic therapy (PDT) has attracted significant attention [1–7]. PTT is an emerging phototherapeutic modality, which triggers photothermal tumor destruction due to localized hyperthermia generated by photoconversion nanoparticles under near-infrared (NIR) light irradiation. While, in PDT, photosensitizers (PSs) generate toxic reactive oxygen species (ROS) under laser irradiation to induce photodynamic tumor killing [8, 9]. Recently, it has been demonstrated that both PTT and PDT can trigger significant immunogenic cell death (ICD) via producing or exposing tumor-associated agents from dead tumor cell residues [10–12]. Tumor cells undergoing ICD can evoke danger-associated molecular patterns (DAMPs) to recruit intratumoral T lymphocytes infiltration as well as an increased ratio of cytotoxic T lymphocytes (CTLs), resulting in the enhanced immune response [13–15]. Therefore, the development of agents with simultaneous PDT and PTT effects is advantageous to trigger significant ICD with better therapeutic efficacy [16, 17].

Heptamethine cyanine dye (IR-780) with a characteristic absorption peak at 780 nm, has been applied in NIR fluorescence or PA imaging [18, 19]. Moreover, because of the excellent light to heat conversion efficiency and ROS production capacity upon NIR laser irradiation, IR-780 was utilized as a multifunctional agent to trigger both PTT and PDT, simultaneously [20–23]. The “Two in One” therapy mediated by IR-780 could trigger the sustained tumor cell killing even at a lower PDT efficacy in solid tumor due to the limited oxygen supply [24–26]. Unfortunately, owing to the strong hydrophobicity and short circulation time in vivo, the further biomedical applications of IR-780 are restricted [27–29]. So, it is highly desirable to develop an IR-780 delivery platform for an efficient tumor theranostics.

**Scheme 1:**
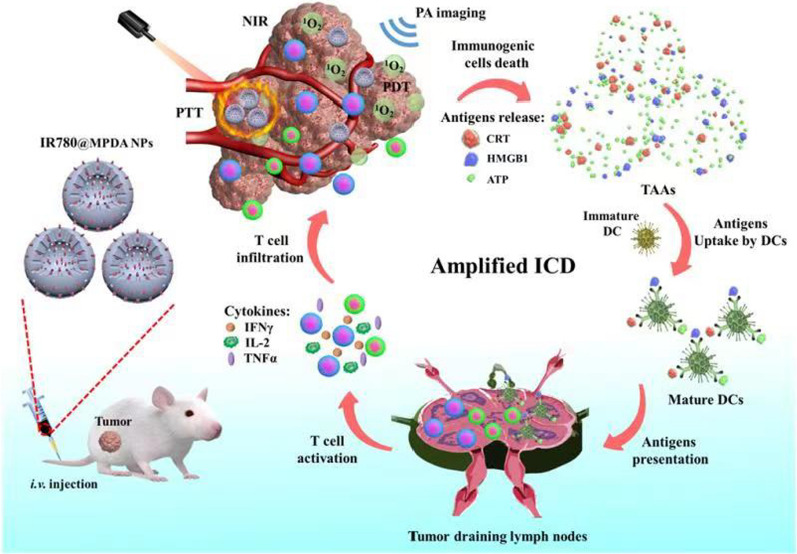
Illustration of PA imaging-guided synergistic phototherapy and immunotherapy triggered by IR-780@MPDA

Herein, IR-780 delivery platform based on mesoporous polydopamine nanoparticles (MPDA) is developed. The hydrophobic IR-780 is loaded onto MPDA through π–π stacking interaction (Additional file 1: Fig. S1). The as-prepared IR-780 loaded MPDA (IR-780@MPDA) exhibited good photothermal conversion ability, and thus are utilized as photoacoustic (PA) contrast agent and photothermal agent for photoacoustic imaging-guided PTT [30–33]. Overall, IR-780@MPDA showed high loading capacity (49.7 wt%), low off-target toxicity, and excellent biocompatibility. The tumor accumulation of IR-780@MPDA was distinctively traced by NIR fluorescence and PA imaging in vivo, respectively. Impressively, an intravenous injection of IR-780@MPDA displayed remarkable tumor growth suppression in 4T1 tumor bearing mice, which is attributed to the combined PDT/PTT along with the elicitation of immune response, triggering enhanced ICD and CTLs activation (Scheme 1).

## Material and methods

### Materials

Dopamine hydrochloride, ammonia aqueous solution (NH_4_OH, 28–30 wt%), ethanol, pluronic F127, 1, 3, 5-trimethylbenzene, and IR-780 were purchased from Sigma-Aldrich (Shanghai, China). Mouse breast cancer cells (4T1) were purchased from American Type Culture Collection (ATCC, Manassas, VA). CD3, CD4, CD8 flow cytometric antibodies and their homotypic antibodies were obtained from BD Biosciences (Shanghai, China). Anti-calreticulin (CRT) antibody was obtained from Abcam (Shanghai, China). The enzyme linked immunosorbent assay (ELISA) kits for the detection of adenosine triphosphate (ATP), high-mobility group box 1 (HMGB1), tumor necrosis factor (TNF-α), interferon γ (IFN-γ), interleukin-2 (IL-2), and 3-(4, 5-dimethylthiazol-2-yl)-2, 5-diphenyltetrazolium bromide (MTT) were obtained from KeyGEN Biotech. Co., Ltd. (Jiangsu, China). Tumor dissociation kit of mouse was purchased from Miltenyi Biotec (Germany). Fetal bovine serum (FBS), trypsin, Roswell Park Memorial Institute 1640 (RPMI 1640) culture medium, and phosphate buffer saline (PBS) were purchased from Gibco/Life Technologies (Shanghai, China). Singlet oxygen sensor green (SOSG) kits were purchased from Invitrogen (Shanghai, China).

### Synthesis of MPDA

Dopamine hydrochloride (0.5 g) and F127 (1.0 g) were added into 100 mL of mixture (water:ethanol = 1:1), and stirred until the powder was well-dissolved. Then, 2.0 mL of trimethylbenzene was slowly injected into the mixture at 500 rpm for 30 min at room temperature. When white turbid liquid was formed, 5.0 mL of NH_4_OH was added drop by drop into the above mixture to initiate the self-polymerization of dopamine polymer nanosphere. After continuous reaction, the formed MPDA were centrifuged at 14,000 rpm for 20 min and washed for 3 times by centrifugation [34].

### Loading capacity of IR-780

For IR-780@MPDA, IR-780 was added into the MPDA solutions and mixed in the dark for 12–24 h. To determine the loading capacity of IR-780, 1 mL of MPDA (100 μg/mL) was dissolved in DI water and mixed with different weight of IR-780 (100, 50, 25, 12.5, 6.25 μg), respectively. The mixture with different weight ratios was stirred continuously for 24 h. Then, all the product solutions were purified by centrifugation at 14,000 rpm for 20 min. After stirring and washing, unconjugated IR-780 was collected and quantified according to the standard curve. The final loading capacity was calculated by the following equation: (weight of total IR-780 − weight of free IR-780)/(weight of total IR-780 − weight of free IR-780 + weight of MPDA).

### Characterization

Spherical morphology and the size of MPDA and IR-780@MPDA were observed by transmission electron microscopy (TEM, JEOL JEM-2100, Japan). The absorption spectra of IR-780, MPDA, and IR-780@MPDA were recorded using an ultraviolet–visible (UV–Vis) spectrophotometer (Lambda 35, PerkinElmer, USA). The hydrodynamic diameter and the surface charge of IR-780, MPDA, and IR-780@MPDA were determined using a Zeta PALS analyzer (Brookhaven Instruments Co., Holtsville, NY, USA). The fluorescence intensity of IR-780 and IR-780@MPDA was observed using an IVIS Lumina XR system (Caliper, Alameda, CA, USA). The fluorescence spectra of IR-780 and IR-780@MPDA were further obtained by fluorescence spectrophotometer (F-4600, HITACHI, Japan). Nitrogen sorption isotherms of the MPDA were carried out, and the surface area was calculated by Brunauer–Emmett–Teller (BET) method. The pore size was calculated using Barrett–Joyner–Halenda (BJH) method. IR-780@MPDA was dispersed in different physiological solutions (H_2_O, saline, RPMI 1640, and FBS) for 24 h, and the UV spectroscopy and DLS analysis were performed to estimate the solubility and stability.

### Photodynamic and photothermal properties

To test the PTT properties, 1 mL IR-780, MPDA, and IR-780@MPDA with equal proportion concentrations were irradiated with an 808 nm wavelength laser at 1 W/cm^2^ for 300 s (Hi-Tech Optoelectronics Co., Ltd., China). Deionized water (1 mL) was used as a control. Similarly, laser power (0, 0.2, 0.5, 1, and 2 W/cm^2^) and concentration (12.5, 25, 50, 100, and 200 μg/mL) dependent photothermal effect were also recorded under 808 nm laser irradiation for 300 s. To further investigate the photostability, 1 mL of 100 μg/mL IR-780@MPDA or 40 μg/mL IR-780 was exposed to the NIR laser at a power density of 1 W/cm^2^ for five laser ON–OFF cycles (ON, 300 s; OFF, 480 s). The corresponding temperature changes during laser irradiation were monitored in real time using an infrared camera (MAGNITY f15F1, Wuhan VST Light & Technology Co., Ltd., China). To determine the photodynamic properties, the generation of singlet oxygen by IR-780, MPDA, and IR-780@MPDA was determined from the fluorescence signal of SOSG (excitation: 504 nm, emission: 525 nm) after irradiation of NIR laser for 1, 3, and 5 min.

Then the singlet oxygen quantum yield of IR-780@MPDA in deionized water was measured using indocyanine green (ICG) as a standard. The same proportional concentration solutions of IR-780@MPDA or ICG were mixed with SOSG (6 μg/mL), respectively. The mixture were prepared and exposed to 808 nm laser irradiation for 0, 15, 30, and 60 s. The UV absorption of ICG (at 780 nm) and the fluorescence of SOSG were measured after each irradiation. Finally, the singlet oxygen quantum yield of IR-780@MPDA was calculated using the following formula:

$$\Phi_{{IR{ - }780@MPDA}} = \Phi_{ICG} K_{{IR{ - }780@MPDA}} A_{ICG} / \, K_{ICG} A_{{IR{ - }780@MPDA}}$$

(Φ_*ICG*_: the singlet quantum yield of ICG, *K*_*IR-780*_: the rate constant of SOSG in the presence of IR-780@MPDA, *K*_*ICG*_: the rate constant of SOSG in the presence of ICG, *A*_*IR-780@MPDA*_: the UV absorption of IR-780@MPDA, *A*_*ICG*_: the UV absorption of ICG).

### Assessment of photothermal conversion efficiency

Aqueous suspensions of MPDA (100 μg/mL, 1 mL), IR-780@MPDA (100 μg/mL, 1 mL), and IR-780 (40 μg/mL, 1 mL) were irradiated by a NIR laser (1 W/cm^2^) for 5 min, and then cooled down naturally. The photothermal conversion efficiency (PTCE) was calculated using the following equation:

$$\eta =\frac{hS \left({T}_{max}-{T}_{amb}\right)-{Q}_{0}}{I\left(1-{10}^{-A}\right)}\times 100\mathrm{\%}$$

(*h*: heat transfer coefficient, *S*: the exposed surface area of a cuvette, *T*_max_: maximum temperature at equilibrium, *T*_amb_: minimum temperature at equilibrium, *Q*_0_: heat absorbed by the container, *I*: incident laser power in W, *A*: absorbance at 780 nm).

When the energy input is equal to the energy dissipated:

$$hS=\frac{\sum {m}_{i}{C}_{p,i}}{{\tau }_{s}}\approx \frac{{m}_{{H}_{2}O}{C}_{{H}_{2}O}}{{\tau }_{s}}$$

(*τ*_*s*_*:* the time constant, $m_{{H_{2} O}}$: the weight of H_2_O, $C_{{H_{2} O}}$: the specific heat capacity of H_2_O).

When the laser is off, the mixture is cooled down:

$$\mathrm{t}=-{\tau }_{s}ln\theta =-{\tau }_{s}ln\frac{T-{T}_{amb}}{{T}_{max}-{T}_{amb}}$$

(t: the time during the cooling stage, *T*: the temperature of mixture at this time).

### Cell culture and cellular uptake

According to the ATCC’s protocol, 4T1 cells were cultured in RPMI 1640 under a 5% CO_2_ atmosphere at 37 °C. When the 4T1 cell concentration reached 70%, cells were incubated with IR-780@MPDA (100 μg/mL) for 8, 12, and 24 h, while, untreated cells were taken as a control. After cells washing and collection, the fluorescence signals from IR-780 were recorded using a flow cytometer (CytoFLEX, Beckman Coulter, USA) and analyzed by FlowJo v10 software (FlowJo LLC, Ashland, OR, USA).

### Detection of cellular ROS

For intracellular ROS detection, 4T1 cells were cultured in six-well plates. When the cells concentrations reached 70–80%, cells were incubated with IR-780, MPDA, and IR-780@MPDA with an equal proportion concentrations for 24 h, and untreated cells were taken as a control. After incubation, the cells in treatment groups were collected to further irradiate with an 808 nm wavelength laser at 1.0 W/cm^2^ for 300 s. All cells were washed, collected, and then incubated with ROS fluorescence probe for 30 min according to the manufacturer’s protocol. The fluorescence signal was recorded by flow cytometer.

### Cell viability assay

For cellular viability, 4T1 cells were seeded in 96-well plates, and incubated with IR-780, MPDA or IR-780@MPDA using the same concentration gradient (0, 25, 50, 100, and 200 μg/mL) for 24 h with/without irradiation (808 nm laser, 1.0 W/cm^2^, 300 s). After washing for three times, the cellular viability was measured by MTT assay.

### Western blot analysis

As the ICD process is accompanied by the release of CRT, western blot analysis was performed to observe the expression of CRT. Cells after various treatments were collected for protein quantitative analysis and protein denaturation. Next, the cellular proteins were separated by electrophoresis and electroblotted to hydrophobic polyvinylidene difluoride (PVDF) membranes. The membranes were then incubated with primary antibodies (anti-CRT, 1:10,000) and secondary antibody. Finally, immunoreactive bands were visualized and quantified using ImageJ software after diaminobenzidine (DAB) coloration. To further evaluate the treatment effects in vivo, western blot analysis was also performed as described above to determine CRT expression in the tumors of each group.

### Biodistribution in vivo

All procedures involving animals were approved by the Institutional Animal Care and Use Committee of Jinling hospital (Nanjing, China). Approximately 5 × 10^6^ 4T1 cells were subcutaneously injected into the right flank of mice to establish animal model. When the tumor sizes reached about 100 mm^3^, a total of 15 mice were used for in vivo imaging, and were divided into three groups (n = 5). Then, mice were injected intravenously with 100 μL of MPDA (1 mg/mL), IR-780@MPDA (1 mg/mL) or 0.4 mg/mL of IR-780, respectively. The in vivo biodistribution was examined after intravenous injection for 0, 2, 4, 6, 8, and 12 h using the IVIS Lumina XR system (excitation: 780 nm, emission: 831 nm) and Vevo LAZR Imaging System (Fujifilm VisualSonics, Toronto, Canada, excitation: 780 nm), respectively. The fluorescence or PA signals of tumors were captured and quantified using Living Image Software.

### Photothermal effect in vivo

Tumor-bearing mice were divided into four groups (n = 5), and injected intravenously with 100 μL PBS, IR-780 (0.4 mg/mL), MPDA (1 mg/mL) or IR-780@MPDA (1 mg/mL) as described above. After the NIR fluorescence and PA imaging guidance, mice were anesthetized and then exposed to an 808 nm wavelength laser at a power density of 1 W/cm^2^ for 300 s. The temperature changes in the tumors were monitored using a MAGNITY f15F1 infrared camera.

### Photodynamic/photothermal therapy in vivo

Tumor-bearing mice were divided into four groups (n = 10). Mice received an intravenous injection of 100 μL PBS, IR-780 (0.4 mg/mL), MPDA (1 mg/mL) or IR-780@MPDA (1 mg/mL), respectively. Then, under NIR fluorescence and PA imaging guidance, mice were irradiated by NIR laser (808 nm, 1 W/cm^2^, and 300 s) after 6 h post-intravenous injection.

### Blood indicators

Tumor-bearing mice were divided into four groups (n = 3). After intravenous injection with 100 μL PBS, IR-780, MPDA or IR-780@MPDA for 48 h as described above. The whole blood of each mouse was drawn to test the important biochemical indicators, such as aspartate aminotransferase (AST), alanine aminotransferase (ALT), alkaline phosphatase (ALP), blood urea nitrogen (BUN), and creatinine (CRE).

### Histopathology

After in vivo tumor therapy, tumor, heart, liver, spleen, lungs, and kidneys from each group were dissected and prepared for pathological sectioning. All the section specimens were stained with hematoxylin and eosin (H&E) staining. Besides, tumor specimens were further subjected to vascular immunohistochemical staining (IHC).

### Cytotoxic T lymphocytes (CTLs) responses

After tumor therapy, tumors were collected from all mice. Then, the total lymphocytes were separated from the mice tumors according to the manufacturer’s instructions. About 10^6^ lymphocytes in each sample were chosen to incubate with CD3, CD4, CD8 antibodies and their homotypic antibodies in the dark for 30 min. The percentage number of cytotoxic T lymphocytes in each group was further recorded by flow cytometer.

### Enzyme linked immunosorbent assay (ELISA)

ATP and HMGB1 are the important release markers of ICD. Whereas, cytokines such as TNF-α, IFN-γ and IL-2, are considered as the crucial markers for CTLs activation-induced systemic immune response. In order to test the marker expression after combined therapy, supernatant after cell treatment or serum samples from the mice of each group were prepared and analyzed by ELISA kits according to the manufacturer’s protocols.

### Statistical analysis

Statistical analysis was performed using the two-sided Student’s t-test for two groups, and two-way analysis of variance for multiple groups using the R Programming Language. Probabilities as *p* < 0.05 (*) and *p* < 0.01 (**) are marked in relevant figures.

## Results and discussion

### Preparation and characterization

**Fig. 1:**
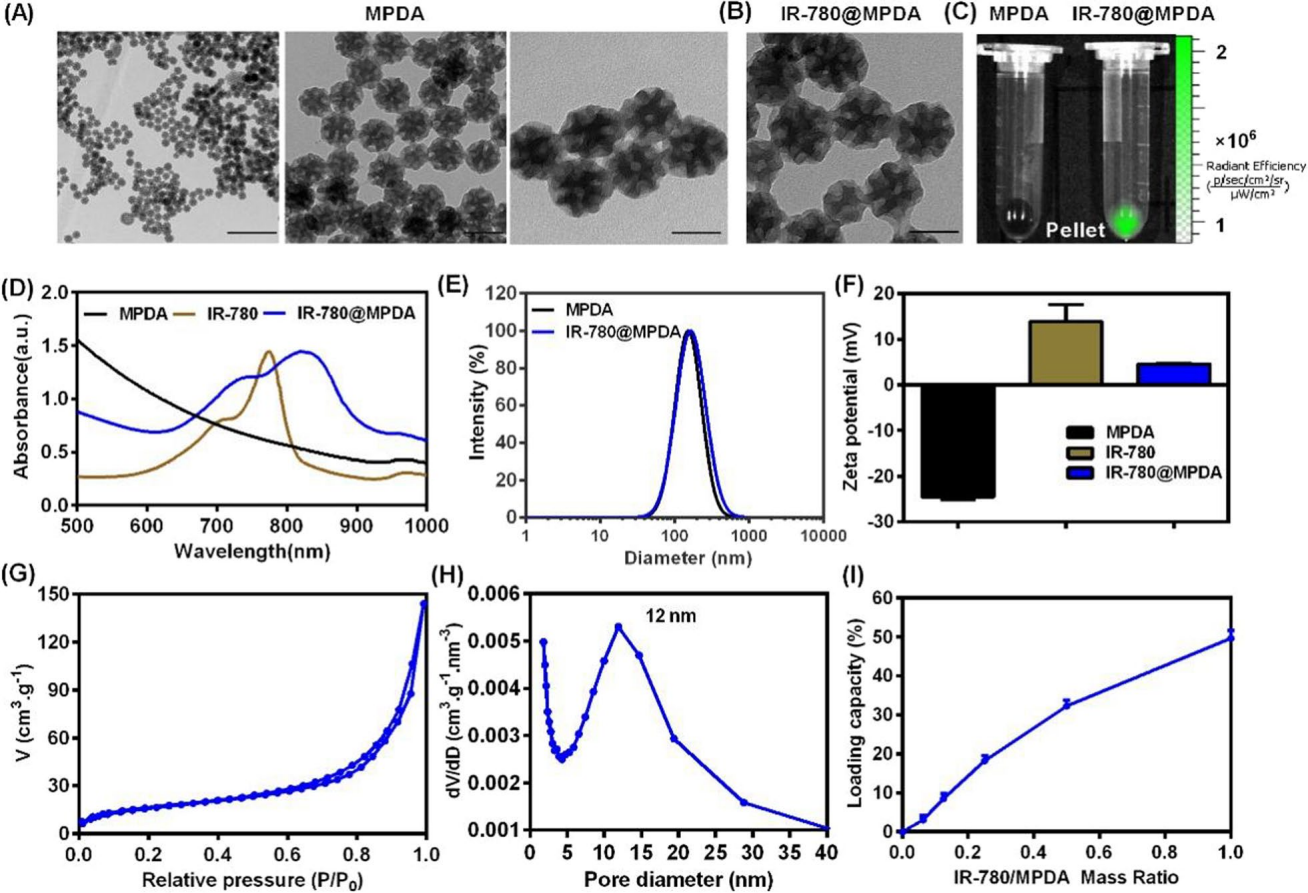
**A** TEM images of MPDA; scale bar 500 nm (left), 100 nm (middle), and 50 nm (right). **B** TEM images of IR-780@MPDA; scale bar 50 nm. **C** The pellet of MPDA and IR-780@MPDA after NIR fluorescence imaging. **D** UV–Vis spectra of MPDA, IR-780, and IR-780@MPDA at the same proportional concentrations. **E** Hydrodynamic diameter of MPDA and IR-780@MPDA. **F** The zeta potential of MPDA, IR-780, and IR-780@MPDA. **G** Nitrogen sorption isotherms of IR-780@MPDA. **H** Pore size distribution curve of the IR-780@MPDA. **I** Loading capacity of MPDA

Transmission electron microscopic (TEM) characterization revealed spherical shape MPDA with an average diameter of 60 nm (Fig. 1A). After IR-780 loading, the size and dispersion of IR-780@MPDA did not change as revealed by TEM (Fig. 1B). The pellet of IR-780@MPDA after centrifugation exhibited fluorescence signals as shown in Fig. 1C, which verified the successful loading of IR-780 onto MPDA. The fluorescence spectra of IR-780 and IR-780@MPDA are presented in Additional file 1: Fig. S2. To further confirm the loading of IR-780, UV–Vis spectra, dynamic light scattering (DLS), and zeta potential measurements were obtained. A characteristic absorption peak of free IR-780 was observed in the absorption spectra of IR-780@MPDA as shown in Fig. 1D, indicating the successful loading of IR-780. On the other hand, after the loading of IR-780, the hydrodynamic diameter of MPDA increased from 152.6 ± 4.8 to 164.8 ± 10.2 nm (Fig. 1E). While, the surface zeta potential shifted from − 24.5 ± 0.6 to 4.55 ± 0.1 mV for IR-780@MPDA, which is attributed to the surface modification of MPDA by positively charged IR-780 (Fig. 1F).

Notably, nitrogen sorption isotherms of the MPDA exhibited a type IV curves in a relative pressure (p/p_0_) ranging from 0.47 to 0.92 with a large hysteresis loop, suggesting that the MPDA had a typical mesoporous architecture with narrow pore size distribution. The surface area and pore volume were calculated to be as high as 866 m^2^/g and 0.4 cm^3^/g, respectively (Fig. 1G). The pore size calculation by BJH method revealed that the MPDA nanoparticles had a uniform mesopores of about 12 nm (Fig. 1H). To investigate the dye loading capacity of MPDA, different concentrations of IR-780 were mixed with MPDA, and the amount of free IR-780 in the supernatants was quantified. As shown in Fig. 1I, the loading capacity of MPDA is directly proportional to the mass ratio (IR-780: MPDA) as the maximum loading capacity (49.7 wt%) was obtained at 1:1 mass ratio. These results suggested that MPDA had a high loading capacity for IR-780. In order to detect the stability, IR-780@MPDA were dispersed in H_2_O, saline, RPMI 1640 and FBS for 24 h. As shown in Additional file 1: Fig. S3A, no particular aggregation was found even after 24 h in different physiological media. Similarly, DLS analysis and UV spectroscopy neither show any change in the hydrophilic diameter nor in absorption spectra over different times (Additional file 1: Fig. S3B–F). These results indicated excellent solubility and stability of IR-780@MPDA in the physiological environment.

### Photodynamic and photothermal properties

**Fig. 2:**
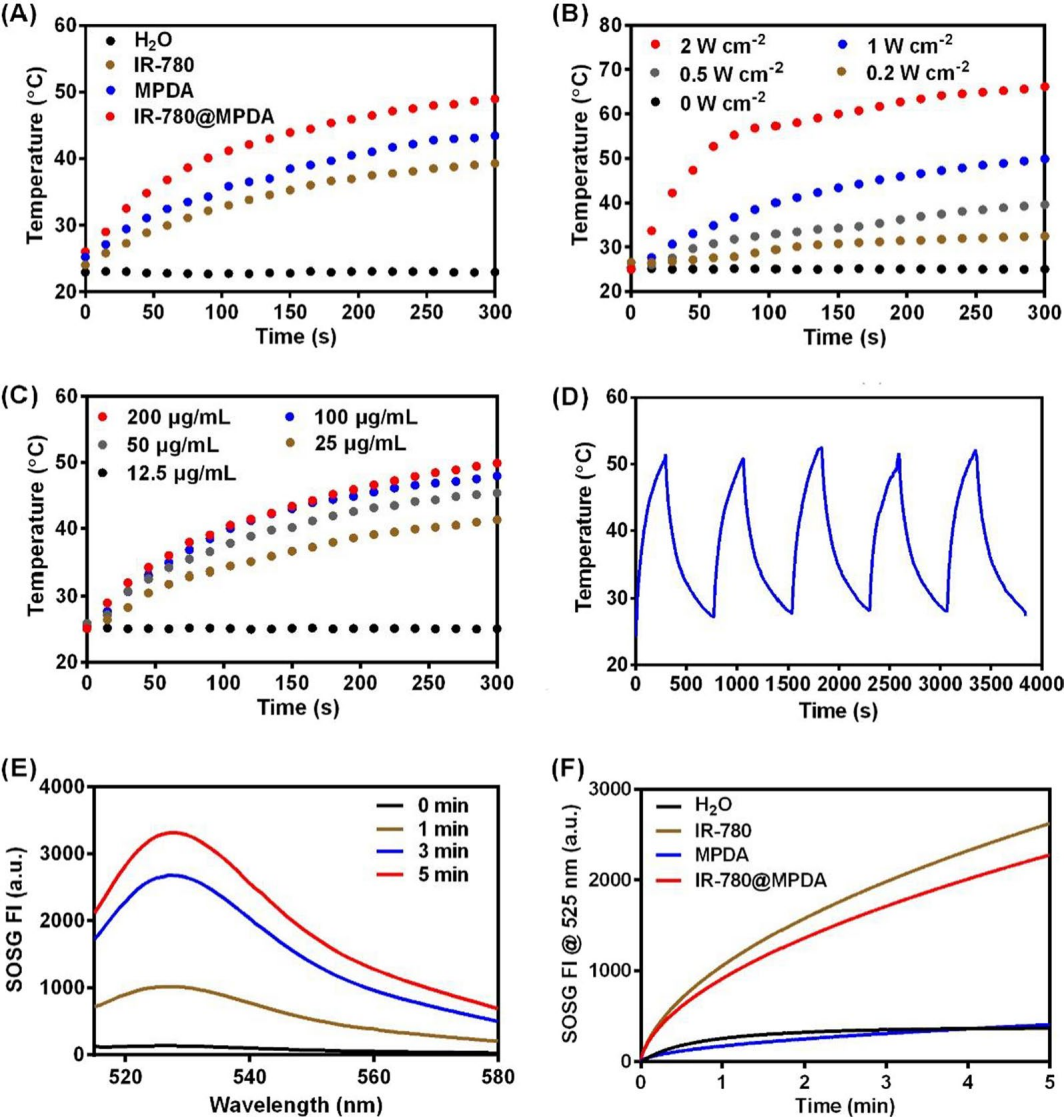
**A** Temperature curves of IR-780, MPDA, and IR-780@MPDA at the same proportional concentrations under laser irradiation (808 nm laser, 1 W/cm^2^, 300 s). H_2_O was taken as a control. **B** Temperature curves of IR-780@MPDA at different irradiation powers (0, 0.2, 0.5, 1, and 2 W/cm^2^). **C** Temperature curves of IR-780@MPDA at different concentrations (12.5, 25, 50, 100, and 200 μg/mL). **D** Photostability of IR-780@MPDA over five cycles of 808 nm laser irradiation at 1 W/cm^2^ for 300 s and cooling for 480 s. **E** SOSG fluorescence intensity (FI) of IR-780@MPDA after different times (1, 3, and 5 min) of irradiation (808 nm, 1 W/cm^2^). **F** SOSG FI of indicated groups at 525 nm under NIR laser irradiation

To investigate the applicability of IR-780@MPDA for simultaneous PDT and PTT, we first tested the photothermal property of IR-780, MPDA, and IR-780@MPDA. As shown in Fig. 2A, water (as a control) did not show any particular temperature increment upon NIR laser irradiation. Whereas, IR-780, MPDA, and IR-780@MPDA demonstrated notable time-dependent temperature elevation. Specifically, an enhanced photothermal activity was recorded by IR-780@MPDA, which is ascribed to the synergistic photothermal effect of IR-780 and MPDA. Moreover, IR-780@MPDA showed laser power-dependent photothermal effect as the maximum temperature (66 °C) was recorded at 2 W/cm^2^ (Fig. 2B). Similarly, concentration-dependent temperature elevation was also noticed as at a concentration of 200 μg/mL, the temperature reached ~ 50 °C under 808 nm laser irradiation (1 W/cm^2^) as shown in Fig. 2C. Such a temperature enhancement within 300 s of NIR laser irradiation was sufficient to trigger localized thermal ablation of tumor cells. Notably, IR-780 showed photothermal degradation under five laser on/off cycles, suggesting photothermal instability and photodegradation (Additional file 1: Fig. S4). Fortunately, the photothermal activity was unchanged even after five consecutive laser on/off cycles, suggesting an excellent photothermal stability of IR-780@MPDA to ensure sustained photothermal tumor cell killing (Fig. 2D). IR-780@MPDA exhibited 54.92% PTCE (*η*), which is superior to both IR-780 (24.11%) and MPDA (42.13%), respectively (Additional file 1: Fig. S5). Notably, the PTCE of IR-780@MPDA is even higher than reported photothermal nanoagents, such as TiO_2_@PDA-Ce6 (32.12%) [35], Au_2_Pt-PEG-Ce6 (31.5%) [36], and PDA-hemoglobin-Ce6 (47.09%) [3].

Along with the photothermal activity, photodynamic properties of IR-780@MPDA were evaluated by determining the ^1^O_2_ production ability via SOSG assay. Figure 2E revealed an obvious increase in ^1^O_2_ generation ability after laser irradiation for 1, 3, and 5 min. The SOSG fluorescence signals of IR-780 and IR-780@MPDA were increased rapidly upon laser irradiation, while no noticeable signal increase was detected in H_2_O or MPDA solution, implying that the ^1^O_2_ generation capacity is solely because of IR-780 molecules rather than MPDA (Fig. 2F). To determine the singlet oxygen quantum yield of IR-780@MPDA, ICG was used as a standard for quantum yield measurement, which was reported to be 0.2 [37]. We found that the UV absorption of IR-780@MPDA from 500 to 1000 nm is 1.82 (*A*_*IR-780@MPDA*_) and 1.83 (*A*_*ICG*_) for ICG (Additional file 1: Fig. S6A, B). Besides, the rate constant of SOSG in the presence of IR-780@MPDA (*K*_*IR-780@MPDA*_) and ICG (*K*_*ICG*_) was 0.0091 and 0.0057, respectively (Additional file 1: Fig. S6C, D). Thus, the calculated singlet oxygen quantum yield of IR-780@MPDA (Φ_*IR-780@MPDA*_) in water was ~ 0.32.

### Cellular uptake and cellular PDT/PTT efficacy

**Fig. 3:**
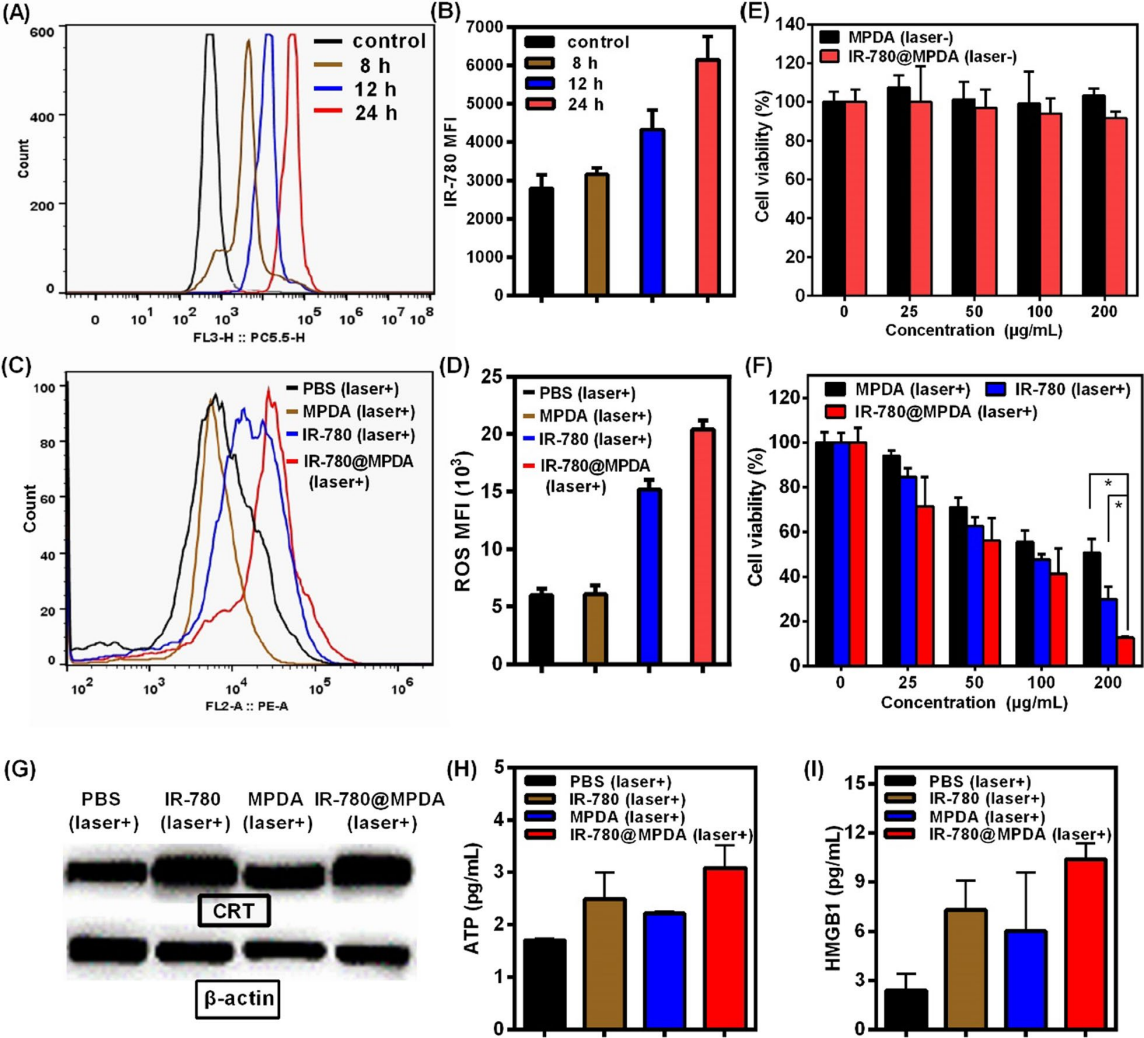
**A** The cellular uptake of 4T1 cells after incubation with IR-780@MPDA for 8, 12 or 24 h. **B** The IR-780 median fluorescence intensity (MFI) at different incubation times. **C** Intracellular ROS generation in control, MPDA, IR-780, and IR-780@MPDA groups under laser irradiation (808 nm, 1 W/cm^2^, 300 s) as indicated. **D** The MFI of ROS in each treatment group. **E** Relative cell viability of 4T1 cells after incubation with different concentrations of MPDA or IR-780@MPDA in dark. **F** Relative cell viability of 4T1 cells under laser irradiation after incubation with different concentrations of MPDA, IR-780 or IR-780@MPDA as indicated. Asterisk * indicated *p* < 0.05. **G** Western blot analysis of CRT expression in vitro. **H** Detection of secreted extracellular ATP. **I** Detection of the released extracellular HMGB1

Prior to the evaluation of combined therapy in vitro, the cellular uptake of IR-780@MPDA was investigated. After cells incubation for different times (8, 12 or 24 h), the fluorescence signal from IR-780 was monitored by flow cytometry. As shown in Fig. 3A, B the fluorescence curves are gradually right-shifted upon an increase in the incubation time, and the maximum fluorescence signal is recorded at 24 h, proving the excellent cellular accumulation abilities of IR-780@MPDA, which is favorable for their combined PDT/PTT therapeutic applications.

Intracellular ROS generation was further evaluated in 4T1 cells using the 2′, 7′-dichlorodihydrofluorescein diacetate (DCFH-DA) probe after incubation with IR-780, MPDA, and IR-780@MPDA in dark or under laser irradiation, respectively. Owing to an excellent intracellular stability, IR-780@MPDA exhibited an enhanced ROS production than free IR-780 or MPDA under NIR irradiation, indicating their high PDT ability at cellular level (Fig. 3C, D). However, without laser excitation, no ROS production was found in each cellular treatment group, suggesting that ROS generation could only be triggered by NIR light activated PDT (Additional file 1: Fig. S7).

The in vitro therapeutic effects against 4T1 tumor cells were further assessed. In dark conditions, more than 80% cellular viability was observed even at a higher concentration (200 μg/mL) of both MPDA and IR-780@MPDA, implying the good in vitro biocompatibility of MPDA and IR-780@MPDA (Fig. 3E). However, under 808 nm laser irradiation for 300 s, a concentration-dependent cellular killing was observed because of the combined PDT and PTT effects (Fig. 3F). The cellular viability of 4T1 cells incubated with IR-780@MPDA was decreased from 71.5 to 12.7% as the concentration increased from 25 to 200 μg/mL. Compared to MPDA and IR-780 alone, IR-780@MPDA exhibited superior therapeutic efficacy especially at a concentration of 200 μg/mL (MPDA vs IR-780@MPDA: *p* = 0.04, IR-780 vs IR-780@MPDA: *p* = 0.04). Such a higher therapeutic killing efficiency by IR-780@MPDA is strongly attributed to the high intracellular delivery of IR-780 and effective combined PDT/PTT therapy in vitro.

The ICD process is usually accompanied by the release of CRT, ATP, and HMGB1 [38, 39]. To investigate whether IR-780@MPDA could trigger ICD after the combined therapy, western blot analysis was first performed to assess the CRT exposure. As shown in Fig. 3G and Additional file 1: Fig. S8, after laser excitation, a noticeable difference in CRT release is observed among the bands in each group as indicated. Compared with the mean gray values (MGV) of the PBS group (67.16 ± 13.63), IR-780@MPDA treatment greatly increased the expression of CRT (MGV = 103.55 ± 16.77) than free IR-780 (MGV = 97.72 ± 17.11) or MPDA (MGV = 86.24 ± 12.42) treatment due to the combined ICD elicitation from PDT and PTT. Similarly, ATP secretion and HMGB1 release in cell supernatant were further assessed by ELISA. Under laser irradiation, IR-780@MPDA treated cells exhibited significantly enhanced expression of ATP and HMGB1, which was remarkably higher than the cells treated with either IR-780 or MPDA, respectively (Fig. 3H, I). All these results proved that IR-780@MPDA could effectively internalize into the tumor cells and elicit significantly amplified ICD for cellular immunity.

### Biodistribution in vivo

**Fig. 4:**
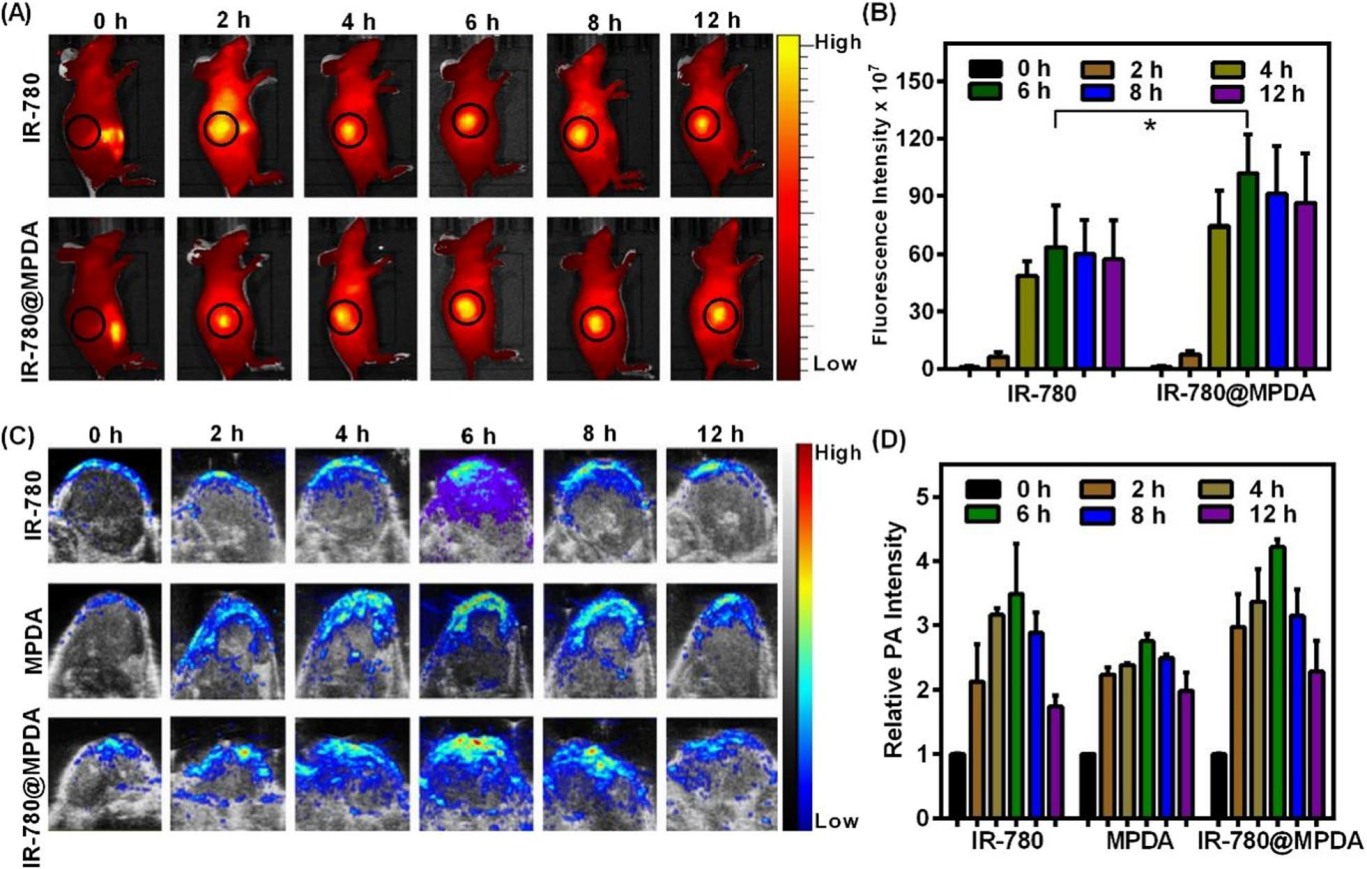
**A** The in vivo biodistribution of IR-780 and IR-780@MPDA at different time points (0, 2, 4, 6, 8, and 12 h) after *i.v.* injection. **B** The fluorescence intensities of tumors at different time points after *i.v.* injection of IR-780 or IR-780@MPDA. Asterisk * indicated *p* < 0.05. **C** PA imaging in vivo at 0, 2, 4, 6, 8, and 12 h by using indicated contrast agents. **D** The relative PA intensities of tumors at different time points after *i.v.* injection of IR-780, MPDA or IR-780@MPDA

The in vivo biodistribution of therapeutic nanoagent is highly important to ensure targeted tumor accumulation and negligible off-target toxicity in vivo [40]. 4T1 mice model were established to trace the biodistribution of intravenously (*i.v.*) injected IR-780 or IR-780@MPDA through an animal optical imaging system. As shown in Fig. 4A, the fluorescence intensities are mainly distributed in the mice abdomen before injection, while, a notable fluorescence signal was seen in the tumor at 2 h post-injection, which got stronger at 6 h, and then decreased at 8 h, especially in the IR-780@MPDA injected group. Specifically, IR-780@MPDA demonstrated 1.6-fold higher accumulation than mice treated with free IR-780 at 6 h post-injection (*p* = 0.04, Fig. 4B), which is possibly because IR-780@MPDA could sustain an effective intratumoral concentration than free IR-780. The enhanced tumor accumulation capability of IR-780@MPDA was highly favorable for tumor therapy in vivo.

Because MPDA and IR-780 possess photothermal activity, the potential of IR-780@MPDA for non-invasive PA imaging in vivo was further evaluated. As shown in Fig. 4C, D, IR-780, MPDA or IR-780@MPDA displayed time-dependent enhanced PA signals, and the maximum PA signal intensity is recorded at 6 h post-injection. Due to the EPR effect, IR-780@MPDA exhibited higher tumor accumulation than free IR-780 and MPDA alone, respectively. The in vivo biodistribution traced by PA imaging was in accordance with the NIR fluorescence imaging. Considering the potential advantages of multimodal imaging, the combined treatment could be guided more precisely to achieve desired therapeutic effects at an optimal treatment time.

### Combined therapy and ICD in vivo

**Fig. 5:**
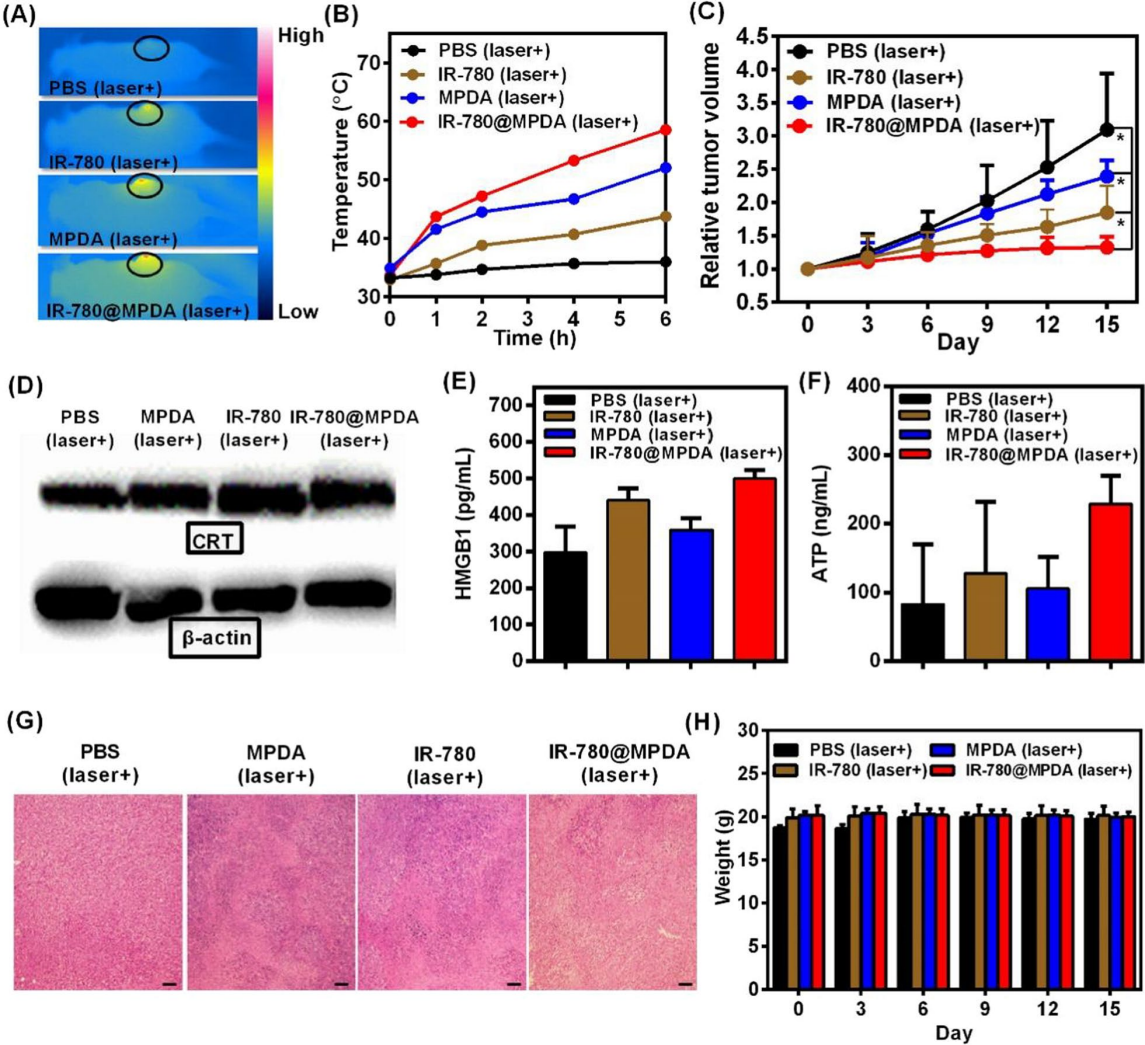
**A** The representative thermal images of tumor-bearing mice after *i.v.* injection of PBS, IR-780, MPDA, and IR-780@MPDA at 6 h. **B** Tumor temperature elevation under laser irradiation (808 nm, 1 W/cm^2^, and 300 s) in mice after *i.v.* injection of PBS, IR-780, MPDA, and IR-780@MPDA at 1, 2, 4, and 6 h. **C** Relative tumor volume of tumor-bearing mice after indicated treatments. The tumor volumes were normalized to their initial sizes. Asterisk * indicated *p* < 0.05. **D** Western blot analysis of CRT expression of tumor after different treatments. **E** Detection of HMGB1 release in vivo. **F** Detection of ATP secretion in vivo. **G** H&E staining of tumors after indicated treatments, scale bars: 100 μm. **H** Changes in the body weight of mice after indicated treatments

To investigate the most effective PTT effect with minimal side effects in vivo, 4T1 tumor-bearing mice were injected intravenously with PBS, IR-780, MPDA or IR-780@MPDA, and then irradiated with 808 nm laser at a power density of 1 W/cm^2^ for 300 s at 1, 2, 4, and 6 h post-injection time points under NIR fluorescence/PA imaging guidance (Fig. 5A, B). Compared to the negligible temperature change in the PBS-treated group, a significant temperature increase was seen in the mice injected with other treatment agents. Whereas, the maximum temperature was recorded in the tumor region at 6 h post-injection because of the maximum tumor enrichment, which was in accordance with the imaging guidance. At 6 h post-irradiation, the temperature increase was about 15, 21, and 27 °C in the groups treated with IR-780, MPDA, and IR-780@MPDA. Notably, due to the tumor-accumulation effect and the low power laser density, there was a minimal injury to the normal skins only around the irradiated tumor areas (Additional file 1: Fig. S9). Hence, IR-780@MPDA exhibited superior treatment effects due to the synergistic phototherapeutic ability of IR-780 and MPDA, respectively.

The combined PDT/PTT therapeutic efficacy in vivo was further evaluated to verify the antitumor performance of IR-780@MPDA. Tumor volume of 4T1 tumor bearing mice was monitored for 15 days (Fig. 5C). Compared to the control group, mice treated with IR-780@MPDA displayed significant suppression of tumor growth. The therapeutic efficacy of IR-780@MPDA was remarkably greater than free IR-780 (*p* = 0.02) and MPDA alone (*p* = 0.02).

In order to investigate the combined phototherapy induced ICD in vivo, the crucial ICD biomarkers like CRT, ATP, and HMGB1 were further determined after different treatments for 2 weeks. As expected, the expression level of CRT was remarkably increased by the treatment of IR-780@MPDA (MGV = 99.47 ± 17.11), which was notably higher than free IR-780 (MGV = 90.10 ± 11.43) or MPDA (MGV = 68.59 ± 15.60) treatment groups (Fig. 5D and Additional file 1: Fig. S10). It is worthwhile to mention that the in vivo findings are in accordance with the western blot analysis. After collecting mice serum from each treatment group, the release of ATP and HMGB1 were detected by ELISA. Figure 5E, F showed the enhanced expression of both ATP and HMGB1 after PDT or PTT alone as compared to the control group, while a significant enhancement was recorded after combined PDT/PTT induced by IR-780@MPDA. All findings proved that IR-780@MPDA could elicit ICD in vivo.

After treatments, H&E staining of tumor sections was performed, which further confirmed that IR-780@MPDA treatment caused more severe necrosis than other treatment agents (Fig. 5G). Moreover, the combined PDT/PTT therapy by IR-780@MPDA inhibited the growth of tumor blood vessels, implying that synergistic phototherapy might influence tumor angiogenesis (Additional file 1: Fig. S11). No apparent weight loss was noticed during the treatment and observation period as shown in Fig. 5H. These findings suggested that mesoporous dopamine delivery nanoplatform exhibited superior synergistic antitumor therapeutic effects.

### Immune response in vivo

**Fig. 6:**
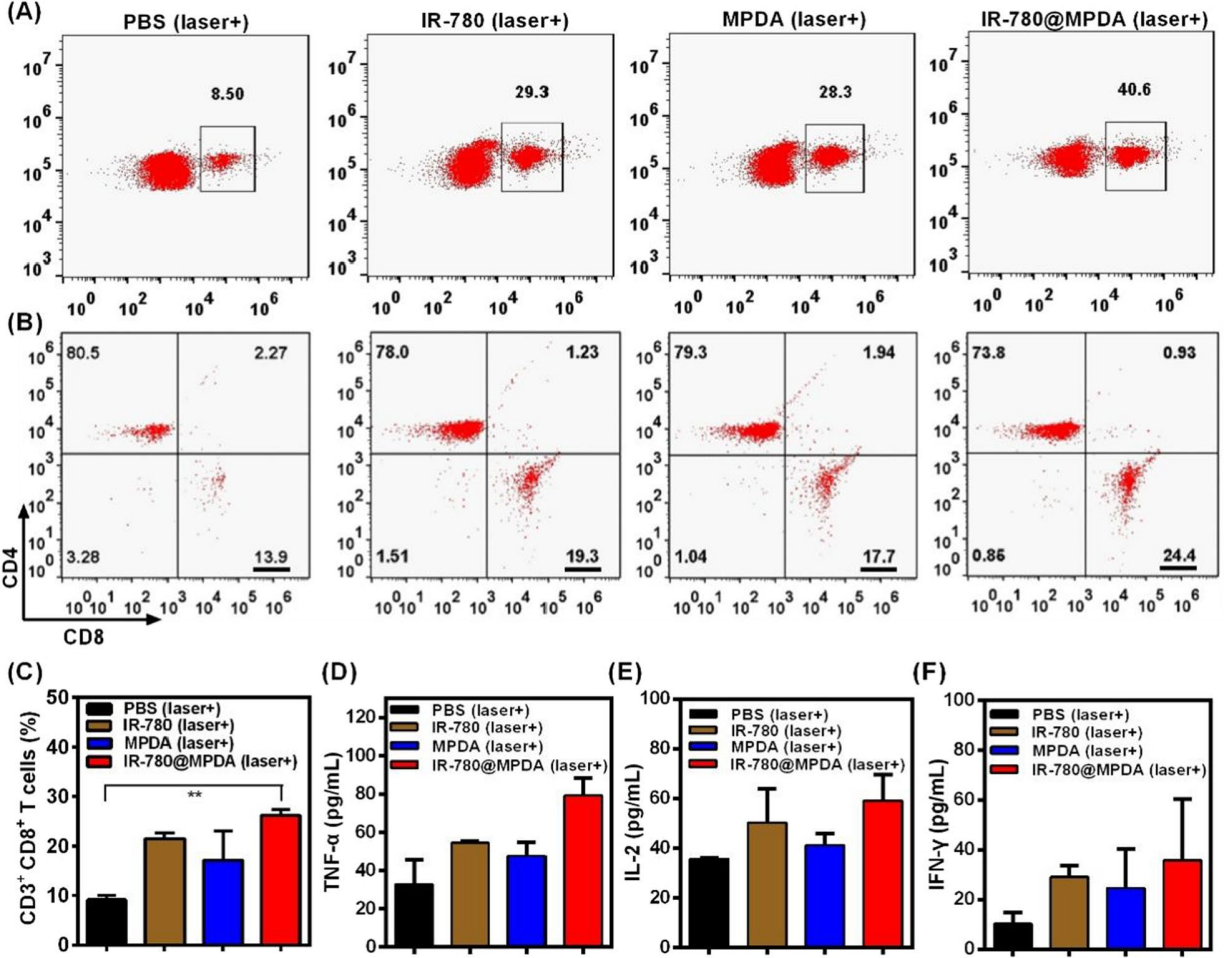
**A** CD3^+^ T cells from tumors were analyzed by flow cytometry after treatments with PBS, IR-780, MPDA or IR-780@MPDA under laser irradiation (808 nm, 1 W/cm^2^, 300 s). **B** Cytotoxic T lymphocytes (CTLs: CD3^+^ CD4^−^ CD8^+^) were analyzed by flow cytometry after different treatments as indicated. **C** Expression analysis of the CTLs after indicated treatments. Asterisk ** indicated *p* < 0.01. Cytokines level, including **D** TNF-α, **E** IFN-γ, and **F** IL-2 in mice serum samples isolated at 15th day after different treatments

Previous studies demonstrated that ICD could generate synergistic immunological effects, resulting in an enhanced recognition of immune cells [41, 42]. To explore the immune stimulatory effects after PDT/PTT induced by IR-780@MPDA, total lymphocytes were extracted from the tumors of each mice at 15th day post-treatment. As shown in Fig. 6A, the frequency of CD3^+^ T cells in IR-780@MPDA treated mice group (40.6%) was significantly higher than IR-780 treated group (29.3%), MPDA treated group (28.3%), and PBS treated group (8.5%), verifying that IR-780@MPDA could evoke greater immunostimulatory effects through the tumor-associated antigens release by PDT/PTT elicited ICD. Moreover, CTLs (CD3^+^ CD4^−^ CD8^+^) are the important subtypes of T lymphocytes and could kill tumor cells directly via immunotherapy. Therefore, the relative frequency of CTLs in CD3^+^ T cells was explored to identify the immune response effects of IR-780@MPDA. As shown in Fig. 6B, IR-780@MPDA triggered significant stimulation of CD8^+^ CTLs (24.4%), which was higher than IR-780 (19.3%), MPDA (17.7%), and PBS (13.9%) after treatments. Quantitative analysis presented in Fig. 6C revealed 2.9-fold percentage of CTLs from IR-780@MPDA than PBS treated group (*p* = 0.001), promoting more lymphocytes to attack tumor cells. Moreover, TNF-α, IL-2 and INF-γ are important indicators of the activation of cellular immunity, and their enhanced expression was found in the group of IR-780@MPDA under laser irradiation, indicating an effective ICD and immune response (Fig. 6D–F) [43]. Taken together, IR-780@MPDA exhibited superior therapeutic effects not only because of the tumor targeting PDT/PTT effects but also by the enhanced immune stimulatory effects.

### Biocompatibility and toxicity evaluation in vivo

**Fig. 7:**
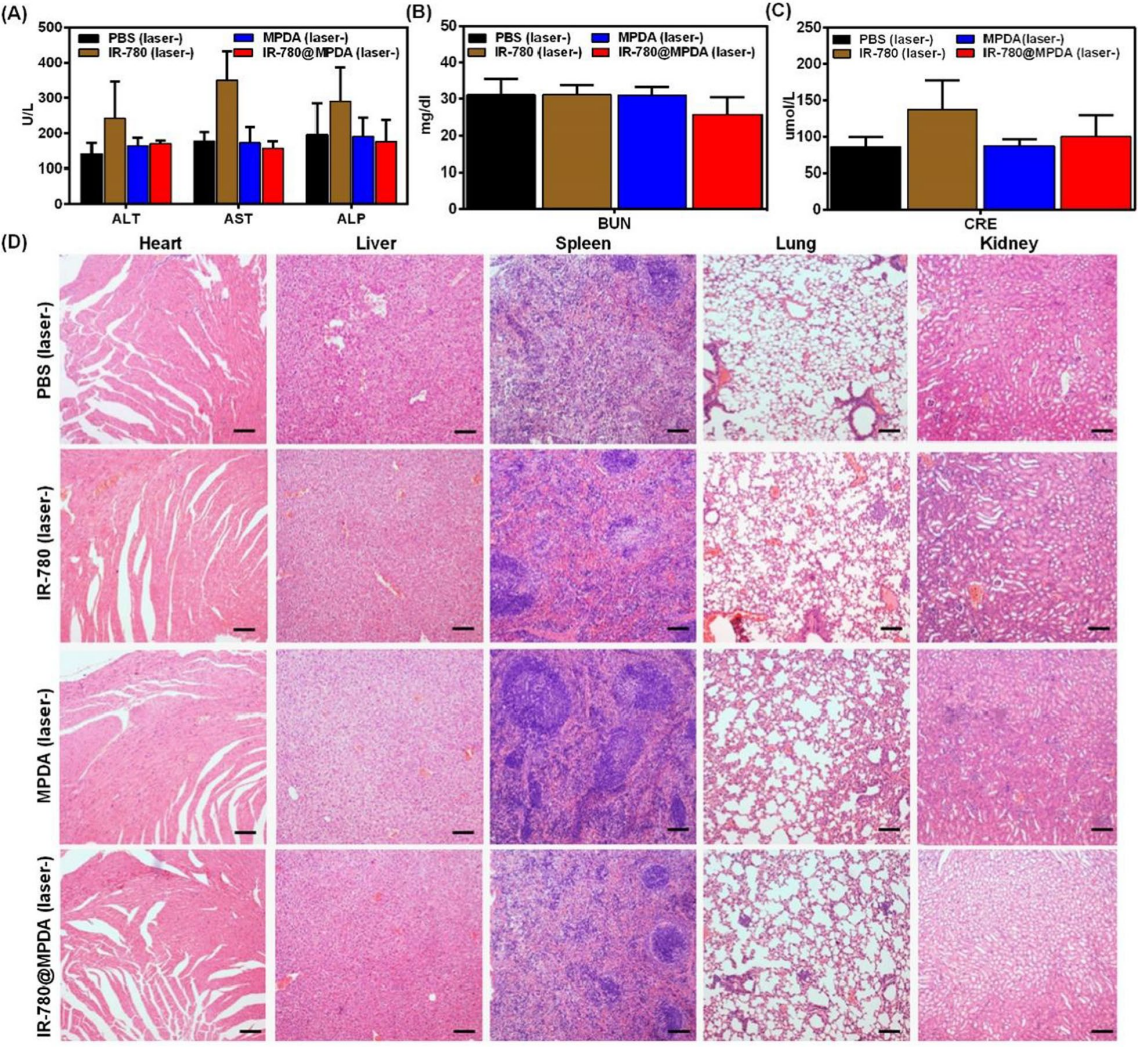
The blood biochemical analysis after *i.v.* injection of PBS, IR-780, MPDA or IR-780@MPDA. Data were obtained for **A** ALT, AST, ALP, **B** BUN, and **C** CRE. **D** H&E staining of the main organs harvested from mice after 6 h post-*i.v.* injection of PBS, IR-780, MPDA or IR-780@MPDA. Scale bars: 100 μm

To determine the safety of IR-780, MPDA or IR-780@MPDA in vivo, healthy mice were chosen and the blood was drawn after 6 h post-*i.v.* injection of the as-mentioned treatment agents. The blood biochemical indicators of liver and kidney functions, including AST, ALT, ALP, BUN, and CRE, were determined. As presented in Fig. 7A–C, compared with the control group (injected with PBS), no significant differences were found between the groups injected with MPDA or IR-780@MPDA, further demonstrating the good biosafety in vivo. However, the expressions of AST, ALT, ALP or CRE in mice injected with free IR-780 were higher. The increase of AST, ALT and ALP reflected liver damage and the possible impairment of heart (Fig. 7A), while an increase in CRE level indicated the poor renal function (Fig. 7C). In addition, H&E staining of the vital organs collected from each group did not reveal an apparent injury except IR-780 group. In IR-780 treated group, the blood vessels of heart, lungs, and liver were expanded with bleeding. Besides, many inflammatory cells were infiltrated in liver and kidneys, while splenic corpuscles were also appeared in spleen (Fig. 7D). These degeneration and inflammation indicated the potential toxicity of free IR-780, whereas, the integration of IR-780 with MPDA resulted in a significantly reduced toxicity in vivo, and thus IR-780@MPDA with excellent biocompatibility and negligible toxicity are highly suitable for clinical transformation.

## Conclusion

In summary, we established “Two in One” tumor collaborative therapy by mesoporous polydopamine delivery nanoplatform loaded with NIR dye IR-780 (IR-780@MPDA). IR-780 can serve as a multifunctional agent for duplex NIR fluorescence/PA imaging-guided tumor PDT and PTT in vivo. After loading onto the MPDA, the biocompatibility, water-solubility, delivery capacities, and potential toxicity of IR-780 were all greatly improved. Meanwhile, due to the combined effect, IR-780@MPDA triggered synergistic targeted phototherapy, resulting in tumor elimination and microvessels inhibition in vivo. Furthermore, the ICD induced by synergistic PDT/PTT caused the activation of CTLs at tumor sites for potential immunotherapeutic response. Such a compact and practical strategy may further be broadened to other solid tumors to improve the therapeutic efficacy.

## Supplementary Information

**Additional file 1: Figure S1. **Schematic design of IR-780@MPDA nanoplatform. **Figure S2. **The fluorescence spectra of IR-780 and IR-780@MPDA. **Figure S3. **(A) The digital photographs of IR-780@MPDA dispersions in H_2_O, saline, RPMI 1640, and FBS at 0 and 24 h. (B) The hydrodynamic diameters of IR-780@MPDA in various physiological solutions at 0, 6, 12, and 24 h. (C–F) UV-Vis spectra of IR-780@MPDA in various physiological solutions as mentioned at 0, 6, 12, and 24 h. **Figure S4. **Photothermal stability of IR-780 over five laser on/off cycles of 808 nm laser irradiation at 1 W/cm^2^ for 300 s and cooling for 480 s. Inset is the digital photographs of IR-780 solution before and after NIR laser irradiation for 300 s. **Figure S5. **(A) Photothermal effect of IR-780@MPDA was recorded under NIR laser irradiation for 5 min and then naturally cooled down. (B) Photothermal effect of MPDA. (C) Photothermal effect of free IR-780. (D) Linear time data versus −ln (θ) obtained from the cooling period of (A). (E) Linear time data versus −ln (θ) obtained from the cooling period of (B). (F) Linear time data versus −ln (θ) obtained from the cooling period of (C). **Figure S6. **(A) The absorption spectrum of IR-780@MPDA. (B) The absorption spectrum of ICG. (C) Linear plot of the increased fluorescence intensity (525 nm) of SOSG in the presence of IR-780@MPDA as the irradiation time. (D) Linear plot of the increased fluorescence intensity (525 nm) of SOSG in the presence of ICG as the irradiation time. **Figure S7. **(A) Intracellular ROS generation in control, MPDA, IR-780, and IR-780@MPDA groups under dark conditions. (B) The ROS median fluorescence intensity (MFI) in each group. **Figure S8.** Mean gray values of CRT bands in each cellular group as indicated. **Figure S9.** Representative photographs of 4T1 tumor bearing mice treated with PBS, IR-780, MPDA or IR-780@MPDA before and after NIR laser irradiation. The black circles indicated the locations of the tumors. **Figure S10.** Mean gray values of CRT bands in each treatment group in vivo. **Figure S11. **Vascular IHC staining of tumors after indicated treatments, scale bars: 100 μm.

## Data Availability

All data used to generate these results is available in the main text and additional information.
