# Supplementary material for: Dye-loaded mesoporous polydopamine nanoparticles for multimodal tumor theranostics with enhanced immunogenic cell death
Source: J Nanobiotechnology. 2021 Nov 17;19:365. doi: 10.1186/s12951-021-01109-7 (PMC8596951; doi:10.1186/s12951-021-01109-7)
Supplement: Supplementary file 1 — Additional file 1: Figure S1. Schematic design of IR-780@MPDA nanoplatform. Figure S2. The fluorescence spectra of IR-780 and IR-780@MPDA. Figure S3. (A) The digital photographs of IR-780@MPDA dispersions in H2O, saline, RPMI 1640, and FBS at 0 and 24 h. (B) The hydrodynamic diameters of IR-780@MPDA in various physiological solutions at 0, 6, 12, and 24 h. (C–F) UV-Vis spectra of IR-780@MPDA in various physiological solutions as mentioned at 0, 6, 12, and 24 h. Figure S4. Photothermal stability of IR-780 over five laser on/off cycles of 808 nm laser irradiation at 1 W/cm2 for 300 s and cooling for 480 s. Inset is the digital photographs of IR-780 solution before and after NIR laser irradiation for 300 s. Figure S5. (A) Photothermal effect of IR-780@MPDA was recorded under NIR laser irradiation for 5 min and then naturally cooled down. (B) Photothermal effect of MPDA. (C) Photothermal effect of free IR-780. (D) Linear time data versus −ln (θ) obtained from the cooling period of (A). (E) Linear time data versus −ln (θ) obtained from the cooling period of (B). (F) Linear time data versus −ln (θ) obtained from the cooling period of (C). Figure S6. (A) The absorption spectrum of IR-780@MPDA. (B) The absorption spectrum of ICG. (C) Linear plot of the increased fluorescence intensity (525 nm) of SOSG in the presence of IR-780@MPDA as the irradiation time. (D) Linear plot of the increased fluorescence intensity (525 nm) of SOSG in the presence of ICG as the irradiation time. Figure S7. (A) Intracellular ROS generation in control, MPDA, IR-780, and IR-780@MPDA groups under dark conditions. (B) The ROS median fluorescence intensity (MFI) in each group. Figure S8. Mean gray values of CRT bands in each cellular group as indicated. Figure S9. Representative photographs of 4T1 tumor bearing mice treated with PBS, IR-780, MPDA or IR-780@MPDA before and after NIR laser irradiation. The black circles indicated the locations of the tumors. Figure S10. Mean gray values of CRT bands in [file 12951_2021_1109_MOESM1_ESM.docx]

**Additional Information**

Dye-loaded mesoporous polydopamine nanoparticles for multimodal tumor theranostics with enhanced immunogenic cell death

Ying Tian^1,2,†^, Muhammad Rizwan Younis^3,†^, Yuxia Tang^1,2,†^, Xiang Liao^1,2^, Gang He^3^, Shouju Wang^1,2^, Zhaogang Teng^4^, Peng Huang^3,^*, Longjiang Zhang^1,2,^*, Guangming Lu^1,2,^*

^1^Department of Medical Imaging, Jinling Hospital, Medical School of Nanjing University, Nanjing, 210002, P.R. China

^2^State Key Laboratory of Analytical Chemistry for Life Science, School of Chemistry and Chemical Engineering, Nanjing University, Nanjing, 210093, P.R. China

^3^Marshall Laboratory of Biomedical Engineering, International Cancer Center, Laboratory of Evolutionary Theranostics (LET), School of Biomedical Engineering, Shenzhen University Health Science Center, Shenzhen, 518060, P. R. China

^4^Key Laboratory for Organic Electronics and Information Displays & Institute of Advanced Materials Nanjing University of Posts & Telecommunications, Nanjing, 210023, P. R. China

**
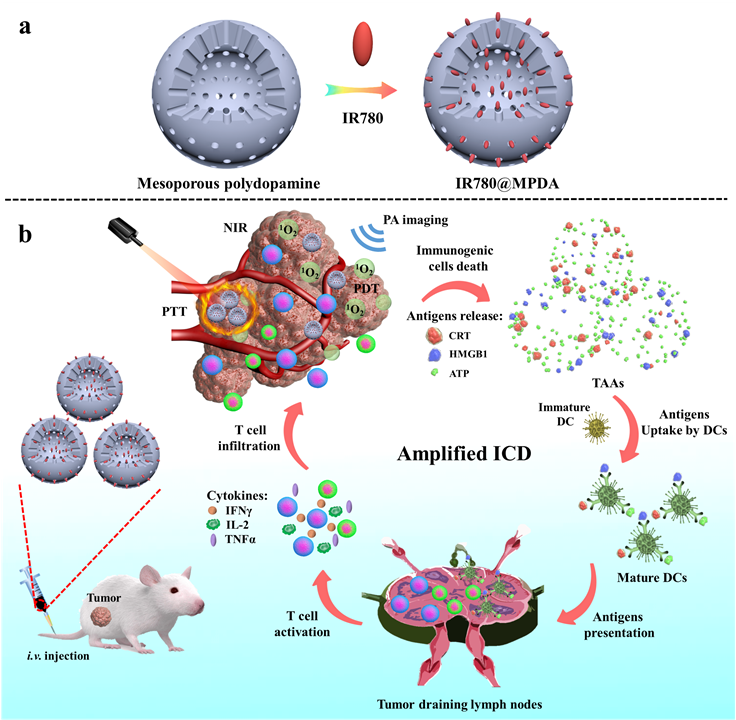
**

**Fig. S1** Schematic design of IR-780@MPDA nanoplatform.


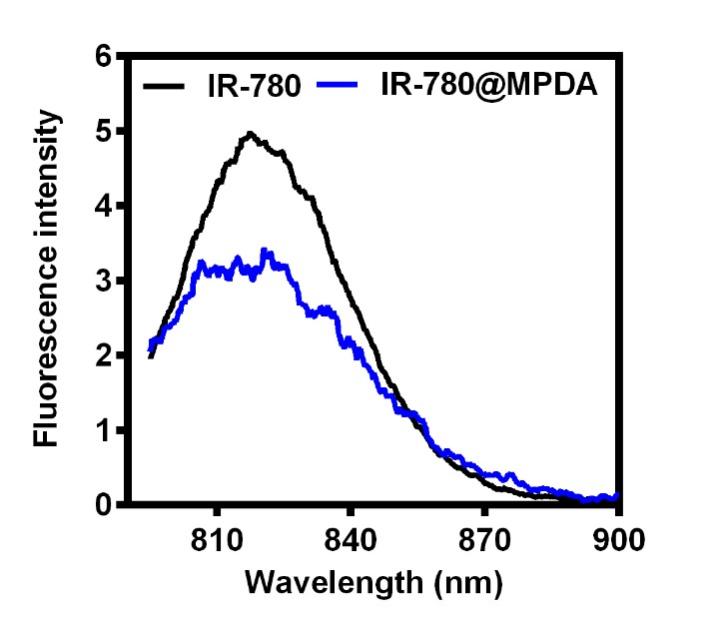


**Fig. S2** The fluorescence spectra of IR-780 and IR-780@MPDA.


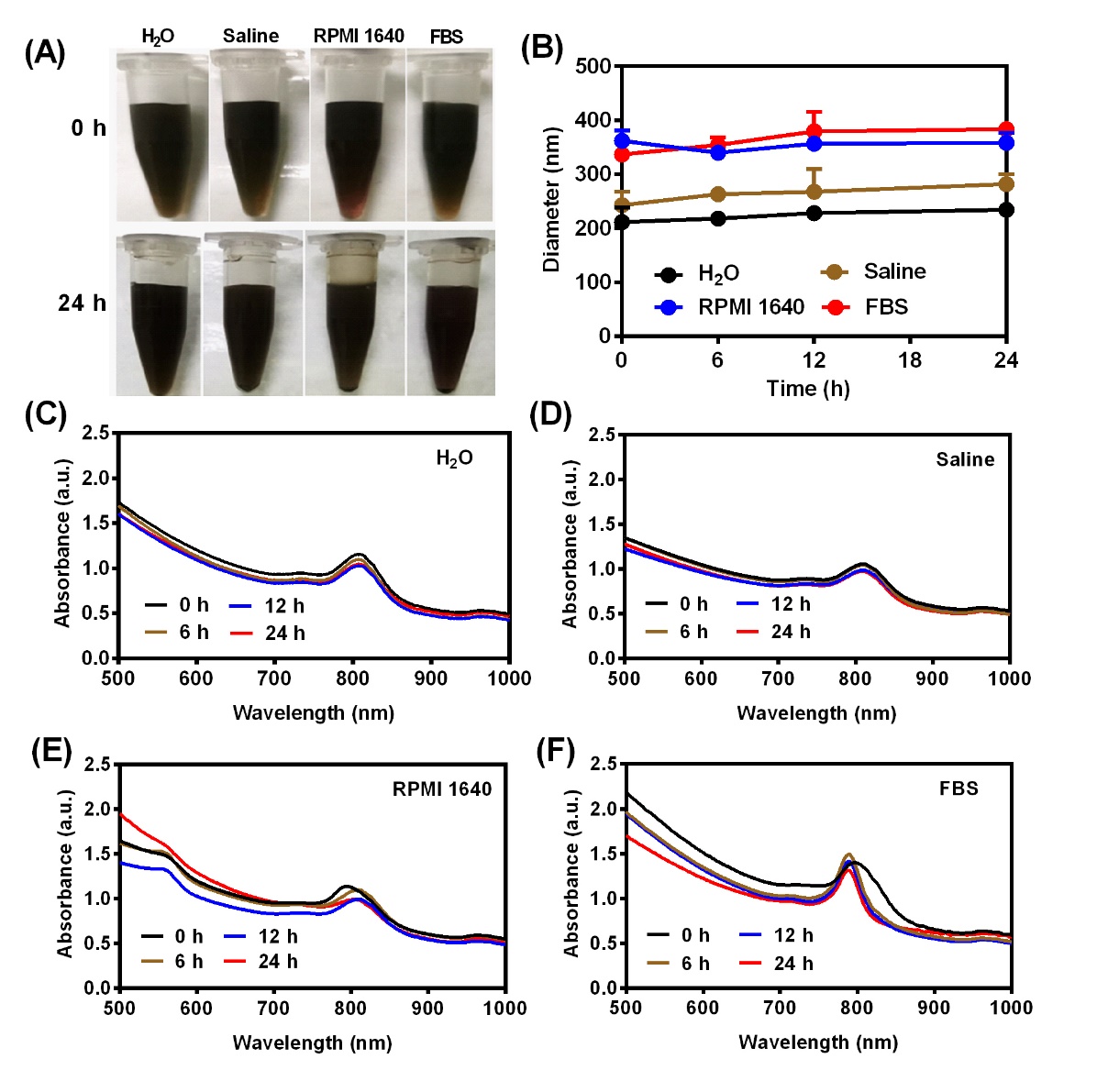


**Fig. S3** (A) The digital photographs of IR-780@MPDA dispersions in H_2_O, saline, RPMI 1640, and FBS at 0 and 24 h. (B) The hydrodynamic diameters of IR-780@MPDA in various physiological solutions at 0, 6, 12, and 24 h. (C-F) UV-Vis spectra of IR-780@MPDA in various physiological solutions as mentioned at 0, 6, 12, and 24 h.

**
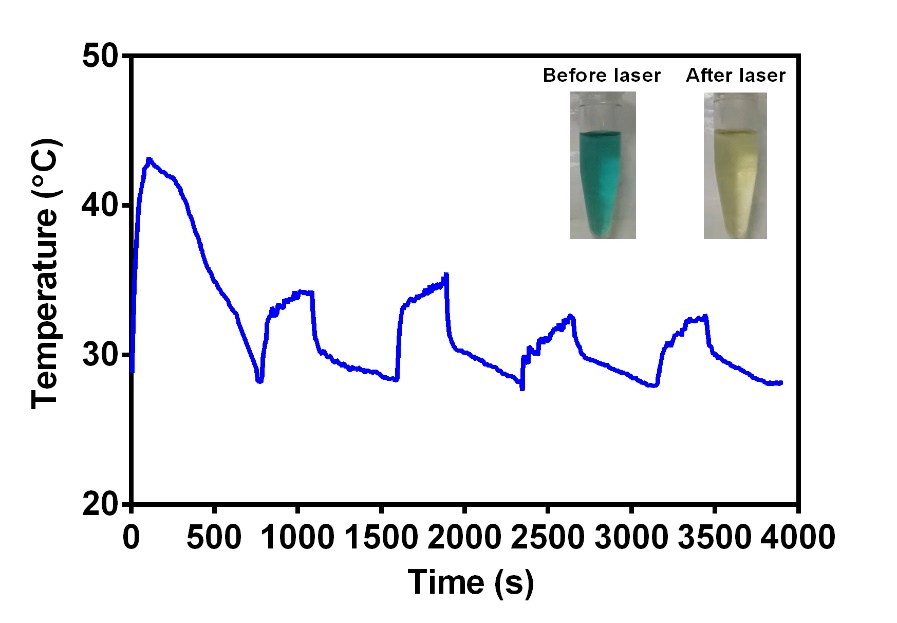
**

**Fig. S4** Photothermal stability of IR-780 over five laser on/off cycles of 808 nm laser irradiation at 1 W cm^-2^ for 300 s and cooling for 480 s. Inset is the digital photographs of IR-780 solution before and after NIR laser irradiation for 300 s.


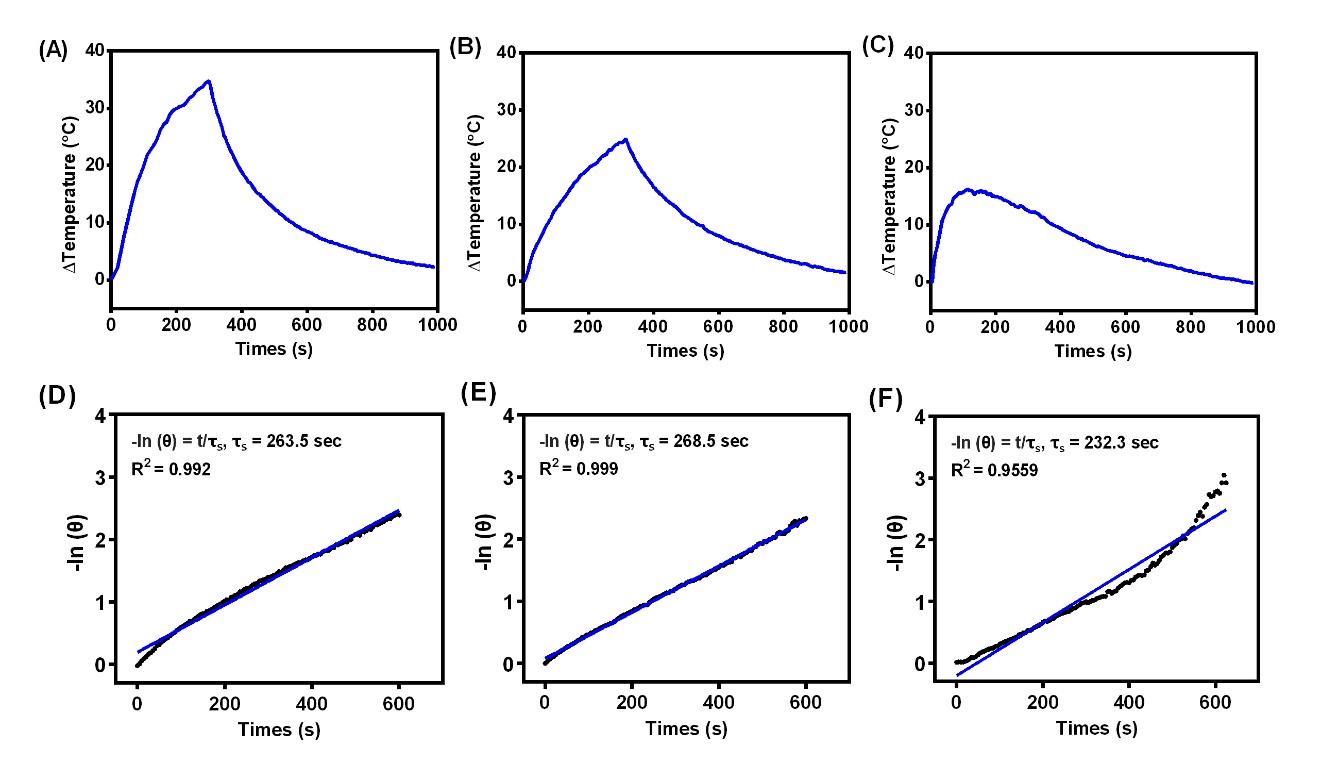


**Fig. S5** (A) Photothermal effect of IR-780@MPDA was recorded under NIR laser irradiation for 5 min and then naturally cooled down. (B) Photothermal effect of MPDA. (C) Photothermal effect of free IR-780. (D) Linear time data versus –ln (θ) obtained from the cooling period of (A). (E) Linear time data versus –ln (θ) obtained from the cooling period of (B). (F) Linear time data versus –ln (θ) obtained from the cooling period of (C).

**
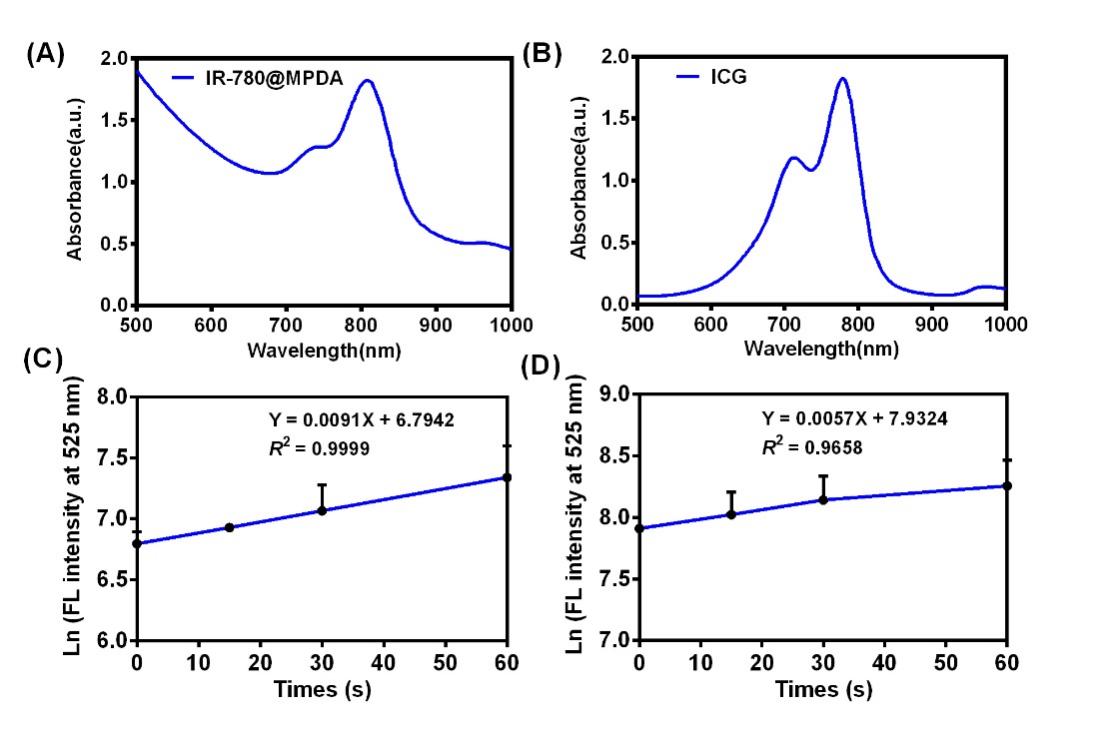
**

**Fig. S6** (A) The absorption spectrum of IR-780@MPDA. (B) The absorption spectrum of ICG. (C) Linear plot of the increased fluorescence intensity (525 nm) of SOSG in the presence of IR-780@MPDA as the irradiation time. (D) Linear plot of the increased fluorescence intensity (525 nm) of SOSG in the presence of ICG as the irradiation time.

**
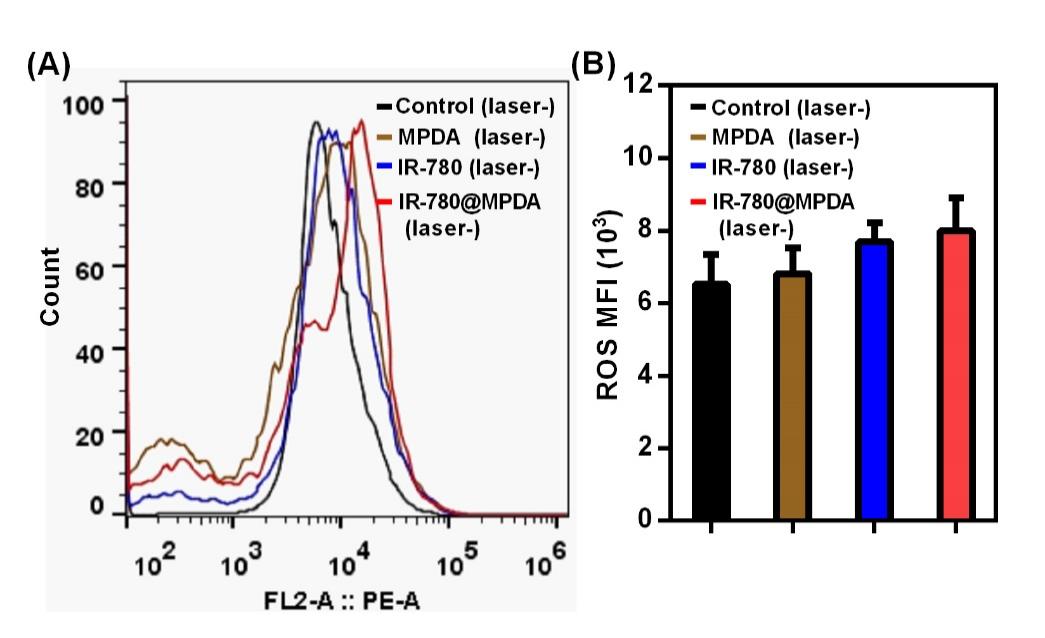
**

**Fig. S7** (A) Intracellular ROS generation in control, MPDA, IR-780, and IR-780@MPDA groups under dark conditions. (B) The ROS median fluorescence intensity (MFI) in each group.


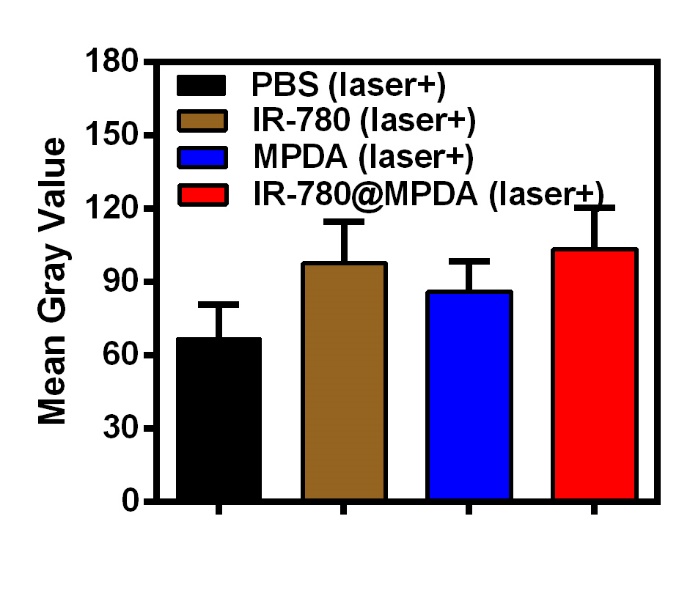


**Fig. S8** Mean gray values of CRT bands in each cellular group as indicated.


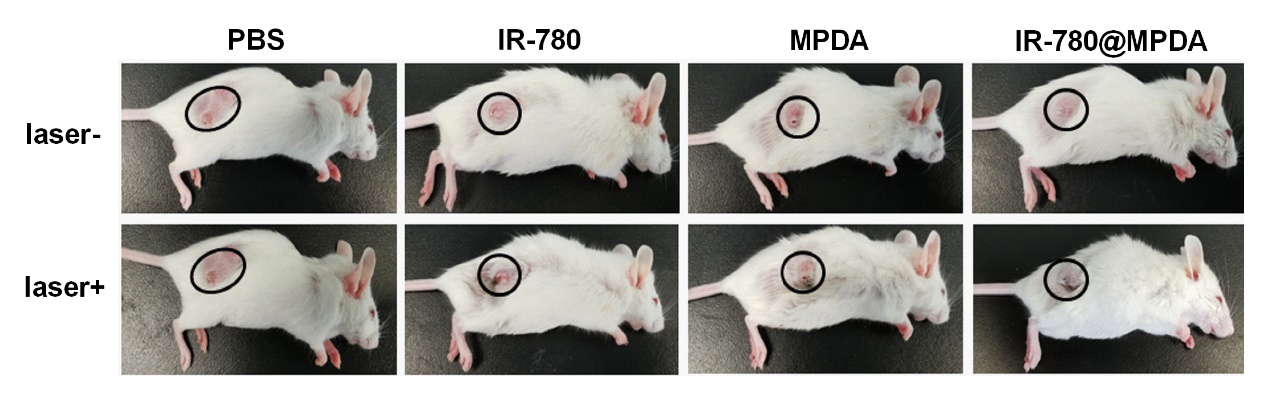


**Fig. S9** Representative photographs of 4T1 tumor bearing mice treated with PBS, IR-780, MPDA or IR-780@MPDA before and after NIR laser irradiation. The black circles indicated the locations of the tumors.


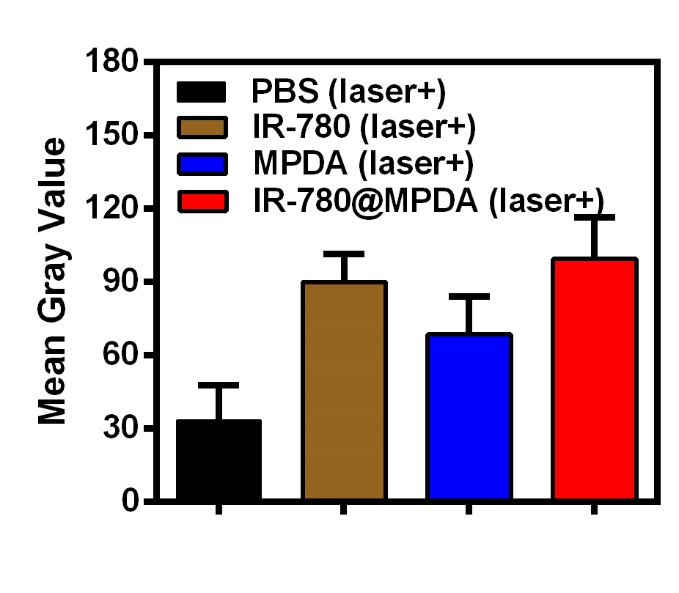


**Fig. S10** Mean gray values of CRT bands in each treatment group *in vivo*.


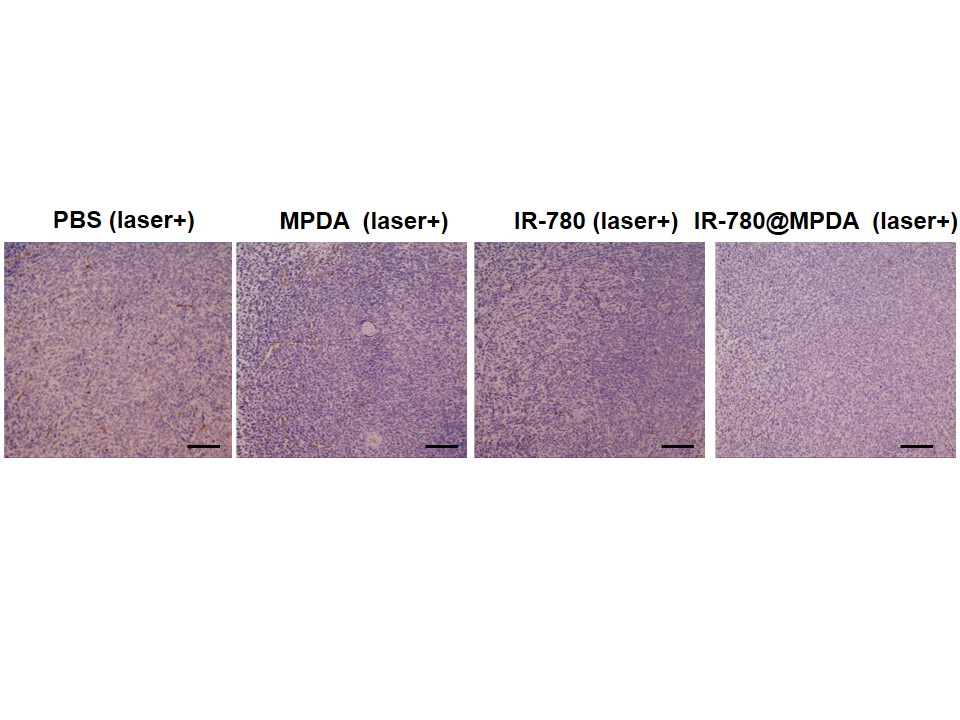


**Fig. S11** Vascular IHC staining of tumors after indicated treatments, scale bars: 100 μm.
